# Mitochondria on the move: Horizontal mitochondrial transfer in disease and health

**DOI:** 10.1083/jcb.202211044

**Published:** 2023-02-16

**Authors:** Lan-Feng Dong, Jakub Rohlena, Renata Zobalova, Zuzana Nahacka, Anne-Marie Rodriguez, Michael V. Berridge, Jiri Neuzil

**Affiliations:** 1 School of Pharmacy and Medical Sciences, Griffith University, Southport, Australia; 2 Institute of Biotechnology, Academy of Sciences of the Czech Republic, Prague-West, Czech Republic; 3 School of Medicine, University of Paris-East, Creteil, France; 4 Malaghan Institute of Medical Research, Wellington, New Zealand; 5 Faculty of Science, Charles University, Prague, Czech Republic; 6 First Faculty of Medicine, Charles University, Prague, Czech Republic

## Abstract

Mammalian genes were long thought to be constrained within somatic cells in most cell types. This concept was challenged recently when cellular organelles including mitochondria were shown to move between mammalian cells in culture via cytoplasmic bridges. Recent research in animals indicates transfer of mitochondria in cancer and during lung injury in vivo, with considerable functional consequences. Since these pioneering discoveries, many studies have confirmed horizontal mitochondrial transfer (HMT) in vivo, and its functional characteristics and consequences have been described. Additional support for this phenomenon has come from phylogenetic studies. Apparently, mitochondrial trafficking between cells occurs more frequently than previously thought and contributes to diverse processes including bioenergetic crosstalk and homeostasis, disease treatment and recovery, and development of resistance to cancer therapy. Here we highlight current knowledge of HMT between cells, focusing primarily on in vivo systems, and contend that this process is not only (patho)physiologically relevant, but also can be exploited for the design of novel therapeutic approaches.

## Teaser

Jiri Neuzil and colleagues review the processes and mechanisms that underlie horizontal mitochondrial transfer (HMT) and the metabolic consequences of HMT in cells.

## Introduction

Mitochondria are essential organelles with multiple functions. As proteobacterial endosymbionts (Gray et al., 1999; Martin et al., 2019), mitochondria retained a part of the original bacterial genome. Mammalian mitochondrial DNA (mtDNA) of ∼15.6 kb codes for 13 mRNAs, whose products are subunits of mitochondrial respiratory complex I (CI), CIII, CIV, and CV, of which CI, CIII, and CIV form the respirasome crucial for CI-dependent respiration (Acin-Perez et al., 2008; Moreno-Lastres et al., 2012; Lapuente-Brun et al., 2013; Gu et al., 2016b). Mitochondrial DNA also encodes 22 tRNAs, 2 rRNAs, and the displacement loop (DLOOP; Falkenberg et al., 2017; Kukat and Larsson, 2013; Kukat et al., 2015; Gustafsson et al., 2016). For proper function, mitochondria need >1,500 proteins, the vast majority of which are encoded by nuclear DNA (nDNA; Liu and Butow, 2006; Neupert and Hermann, 2007; Ryan and Hoogenraad, 2007).

A prominent feature of mtDNA is its heteroplasmy (more than one mitochondrial genotype) mediated by tissue-specific non-random segregation (Burgstaller et al., 2014). Heteroplasmic variance modulates the number of pathological cells in a tissue (Aryaman et al., 2019b). Most eukaryotic cells carry multiple mtDNA copies, and the sequence of each mtDNA molecule can vary. This results in intracellular mitochondrial heterogeneity, which can lead to intercellular mitochondrial heterogeneity (Aryaman et al., 2019a). An intriguing paper proposed that exchange of mitochondria between cells helps maintain balanced heteroplasmy (Jayaprakash et al., 2015). This can be reconciled with the notion that for a particular phenotype, relevant heteroplasmy needs an elevated number of mtDNA copies with this genotype (Picard et al., 2014; Stewart and Chinnery, 2021).

Mitochondria have been increasingly linked to metabolic reprogramming and differentiation (Buck et al., 2016; Sykes et al., 2016; Seo et al., 2018). Mitochondrial function is associated with maintaining and dictating stem cell fate, and this plays a role in metabolic programming during quiescence, activation, self-renewal, proliferation, and differentiation (Bhattacharya and Scimè, 2020). It was found that mitochondrial Akt signaling modulates somatic cell reprogramming (Chen et al., 2019), and there is increasing understanding that mtDNA damage and loss of mitochondrial genome integrity play a critical role in the development of both severe early-onset maladies and chronic age-related diseases (Petros et al., 2005; Moreno-Loshuertos et al., 2006; Schon et al., 2012; Guha et al., 2014; Sharma and Sampath, 2019).

Although the role of mitochondria in cell biology and pathophysiology has been studied and is relatively well established, novel properties of mitochondria and their regulation keep emerging. For example, it has been shown that not all eukaryotes contain mitochondria, with metabolic function being at least in part maintained by complementary systems (Karnkowska et al., 2016). Mitochondria have been long considered autonomous, undergoing cytoplasmic inheritance (Birky, 2001). It is now apparent that there is a close relationship between mtDNA and the nucleus, including synchronized translation of nDNA- and mtDNA-encoded genes (Couvillion et al., 2016; Latorre-Pellicer et al., 2019). Regulation of mtDNA and mitochondrial proteins by epigenetic changes and post-translational modifications facilitate crosstalk between the nucleus and mitochondria that leads to maintenance of cellular homeostasis (Sharma et al., 2019). Consistent with this, mitochondria move to daughter cells during mitosis along microtubular structures (Kanfer et al., 2015).

A prominent place among recent intriguing findings is the identification of horizontal mitochondrial transfer (HMT) with their DNA payload between mammalian cells in vivo in a tumor setting (Tan et al., 2015; Berridge et al., 2015; Dong et al., 2017) and during lung injury (Islam et al., 2012; Ahmad et al., 2014), resulting in respiratory recovery. In the lung studies, mtDNA transfer was assumed but not directly demonstrated, while the cancer studies provide direct evidence for transfer of mtDNA based on distinct polymorphisms (Tan et al., 2015). The cancer studies used syngeneic tumors in transgenic mice with a mitochondria-associated fluorescence protein (su9DsRed2; Dong et al., 2017), while in mouse models of lung injury, the animals were injected with mesenchymal stem cells (MSCs) containing mitotracker-labeled mitochondria (Islam et al., 2012; Ahmad et al., 2014).

Prior to these studies, horizontal transfer of genes in eukaryotes had been considered rare (Keeling and Palmer, 2008), known only in lower eukaryotes (Gladyshev et al., 2008) and plants (Bergthorsson et al., 2003), promoting their biochemical diversification (Boschetti et al., 2012). In mammalian systems, there was evidence for intercellular trafficking of mitochondria in vitro (Rustom et al., 2004), but whether this occurred in vivo and had functional significance remained unclear (Rogers and Bhattacharya, 2013). The four cancer/lung studies discussed above made a strong case for HMT in vivo and for its relevance in pathological settings, and this was supported by phylogenetic evidence for intercellular transfer of mtDNA in canine transmissible venereal tumors (CTVT; Strakova et al., 2016; Strakova et al., 2020).

This Review highlights recent evidence of HMT between cells in vitro and, in particular, in vivo. The different possible modes of HMT, cellular and molecular mechanisms underlying each type of HMT, and metabolic consequences of HMT-related events in physiological and pathological settings linked to pervasive plasticity and therapeutic potential will be discussed.

## Discovery of HMT

Intercellular communication is essential for maintaining homeostasis in multicellular organisms. The modes of cell-to-cell communication are diverse, including entry of various signaling “entities” across the cell membrane (Conner and Schmid, 2003), intercellular junctions (Dejana, 2004), or extracellular vesicles (exosomes, ectosomes, and microvesicles; Stoorvogel et al., 2002; Boelens et al., 2014; Cocucci and Meldolesi, 2015; Becker et al., 2016; Maas et al., 2017; Kumar et al., 2021). The role of such intercellular signaling has been shown in physiological and pathological context, and its therapeutic potential has been highlighted (EL Andaloussi et al., 2013). Interestingly, extracellular vesicles can carry across genetic material, i.e., microRNAs, that modulate the function of the recipient cells (Mittelbrunn and Sánchez-Madrid, 2012; Tomasetti et al., 2017).

A landmark paper defined the so-called “highways for intercellular organelle transport” (Rustom et al., 2004). This was based on the breakthrough discovery of a nanotubular network mediating long- and short-distance communication between cells via transient filamentous membrane protrusions that connect cytoplasm of neighboring or distant cells. These interconnections between cultured cells were shown to allow cell-to-cell movement of intracellular material. The authors coined the term “tunneling nanotubes” (TNTs) for this novel means of intercellular communication. They found that TNTs form between cells of different types. Transfer of cellular material via TNTs was inhibited by latrunculin B, pointing to the role of actin. TNTs were then studied in more detail and have been shown to carry across various cargo. This includes calcium ions (Watkins and Salter, 2005; Smith et al., 2011), bacteria (Onfelt et al., 2006), prions (Zhu et al., 2015; Gousset et al., 2009; Gousset and Zurzolo, 2009), or viruses (Rogers and Bhattacharya, 2013), as well as proteins (Reichert et al., 2016), as found in co-culture studies in vitro. The first report on viral transmission via TNTs involved the spread of human immunodeficiency virus from infected T cells to healthy cells (Sowinski et al., 2008). TNTs were also found to contribute to intercellular transfer of herpesvirus between live cells (Panasiuk et al., 2018) and to the spread of other types of viruses, including SARS-CoV-2 (Tiwari et al., 2021).

TNTs have been reported to translocate various organelles, including endosomes (Rustom et al., 2004; Bukoreshtliev et al., 2009; Wang et al., 2011), ER (Kadiu and Gendelman, 2011a; Kadiu and Gendelman, 2011b), Golgi/ER (Wang et al., 2011), lysosomes (Rustom et al., 2004; Onfelt et al., 2006; Abounit et al., 2016), melanosomes (Gerdes et al., 2007), and mitochondria (Tavi et al., 2010; Koyanagi et al., 2005; Spees et al., 2006; Acquistapace et al., 2011). While other means of HMT between cells have also been identified, TNTs remain a major transport route. The types of cells that “donate” and “import” mitochondria are numerous. Transfer may be homotypic or heterotypic, i.e., donor and acceptor cells are of the same type or of different types, respectively.

Donor cells are often MSCs (Islam et al., 2012; Ahmad et al., 2014; Spees et al., 2006; Otsu et al., 2009; Plotnikov et al., 2008; Boukelmoune et al., 2018). Other types of donor cells, i.e., fibroblasts (Marlein et al., 2017; Ippolito et al., 2019), astrocytes (Sun et al., 2019), hematopoietic cells (Golan et al., 2020), cardiomyocytes (CMs; Nicolas-Avila et al., 2020), or adipocytes (Brestoff et al., 2021; Crewe et al., 2021), have also been reported. Acceptor cells are also of various types, including epithelial cells (Konari et al., 2019), endothelial cells (ECs; D’Souza et al., 2021), CMs (Marti Gutierrez et al., 2022), neuronal cells (Babenko et al., 2018; Li et al., 2019), neural stem cells (Boukelmoune et al., 2018), T lymphocytes (Hough et al., 2018), cancer cells (Spees et al., 2006; Marlein et al., 2017; Moschoi et al., 2016), and macrophages (Brestoff et al., 2021; Jackson et al., 2016a; Jackson et al., 2016b). Intercellular HMT has been reported under both pathological and physiological situations (Table 1).

**Table 1.** Overview of donor/acceptor cells (types of cells) involved in mitochondrial transfer

Donor cells	Acceptor cells	Type of transfer	References
Rat neuroendocrine pheochromocytoma cells	Rat neuroendocrine pheochromocytoma cells	TNTs	Rustom et al., 2004
Human embryonic kidney cells	Human embryonic kidney cells	TNTs	Rustom et al., 2004
Normal rat kidney cells	Normal rat kidney cells	TNTs	Rustom et al., 2004
Rat neonatal CMs	Human endothelial progenitor cells	Nanotubes	Koyanagi et al., 2005
Human macrophage	Human macrophages	TNTs	Onfelt et al., 2006
Human MSCs	Human alveolar adenocarcinoma cells	TNTs	Spees et al., 2006
Human skin fibroblast	Human alveolar adenocarcinoma cells	TNTs	Spees et al., 2006
Human MSCs	Rat CM	Nanotubes	Plotnikov et al., 2008
Rat MSCs	Rat lung ECs	Cx43-based intercellular gap junctional communication	Otsu et al., 2009
MSCs	Rat cardiomyoblasts	Nanotubes	Cselenyak et al., 2010
Rat kidney renal tubular cells	Human MSCs	TNTs and gap junctions	Plotnikov et al., 2010
Human MSCs	Rat kidney renal tubular cells	TNTs and gap junctions	Plotnikov et al., 2010
Mouse endothelial progenitor cells	Human umbilical vein ECs	TNTs	Yasuda et al., 2010
Human adipose-derived stem cells	Mouse CMs	Partial cell fusion	Acquistapace et al., 2011
Human proximal tubular epithelial cells	Human proximal tubular epithelial cells	TNT-like structures	Domhan et al., 2011
Rat ventricular CMs	Rat fibroblasts	Nanotubes	He et al., 2011
Rat ventricular CMs	Rat ventricular CMs	Nanotubes	He et al., 2011
Rat fibroblasts	Rat fibroblasts	Nanotubes	He et al., 2011
Rat fibroblasts	Rat ventricular CMs	Nanotubes	He et al., 2011
Rat hippocampal astrocytes	Rat hippocampal astrocytes	TNTs	Wang et al., 2011
Human MSCs	Human osteosarcoma cells	Partial cell fusion	Cho et al., 2012
Mouse BM-derived stromal cells	Mouse BM-derived stromal cells	Cx43-containing gap junctional channels, nanotubes, MVs	Islam et al., 2012
Human BM-derived stromal cells	Human BM-derived stromal cells	Cx43-containing gap junctional channels, nanotubes, MVs	Islam et al., 2012
Mouse BM-derived stromal cells*	Mouse alveolar epithelial cell*	Cx43-containing gap junctional channels, nanotubes, MVs	Islam et al., 2012
Human BM-derived stromal cells*	Mouse alveolar epithelial cell*	Cx43-containing gap junctional channels, nanotubes, MVs	Islam et al., 2012
Human pleural mesothelioma cells	Human pleural mesothelioma cells	TNTs	Lou et al., 2012
Human MSCs	Human vascular smooth muscle cells	TNTs	Vallabhaneni et al., 2012
Human vascular smooth muscle cells	Human MSCs	TNTs	Vallabhaneni et al., 2012
Human retinal pigment epithelial cells	Human retinal pigment epithelial cells	TNTs	Witting et al., 2012)
Human ECs	Human ovarian cancer cells	TNTs	Pasquier et al., 2013
Human ECs	Human breast cancer cells	TNTs	Pasquier et al., 2013
MSC*	Bronchial epithelial cells*	TNTs	Ahmad et al., 2014
Platelets	Neutrophils	ECVs	Boudreau et al., 2014
Murine neuronal cells*	Murine glial cells*	Protrusions	Davis et al., 2014
MSCs*	Lung epithelial cells*	TNTs	Li et al., 2014
MSCs	Human umbilical vein ECs	TNTs	Li et al., 2014
Human multipotent MSCs	Rat neuronal cells	Intercellular contacts	Babenko et al., 2015
Human multipotent MSCs	Rat glial cells	Intercellular contacts	Babenko et al., 2015
Breast cancer cells	Breast cancer cells	n.s.	Jayaprakash et al., 2015
Normal fibroblast cells	Normal fibroblast cells	n.s.	Jayaprakash et al., 2015
Human MSCs	Macrophages	MVs	Phinney et al., 2015
Human MSCs*	Macrophages*	MVs	Phinney et al., 2015
Pheochromocytoma cells	Pheochromocytoma cells	TNTs	Okafo et al., 2017
MSCs	Rat cardiomyoblasts	TNTs	Han et al., 2016
Astrocytes*	Neuronal cells*	Extracellular mitochondrial particles	Hayakawa et al., 2016
MSCs*	Lung alveolar macrophages*	TNTs	Jackson et al., 2016a, b
MSCs	Corneal epithelial cells	TNTs	Jiang et al., 2016
Mouse sister germ cells	Mouse oocytes	Microtubes	Lei and Spradling, 2016
BM stromal cells	Acute myeloid leukemic cells	Endocytosis	Moschoi et al., 2016
BM stromal cells*	Acute myeloid leukemic cells*	Endocytosis	Moschoi et al., 2016
MSCs*	Murine CMs*	TNTs	Zhang et al., 2016
MSCs	Murine CMs	TNTs	Zhang et al., 2016
MSCs	MERRF cybrid cells	n.s.	Chuang et al., 2017
Malignant urothelial carcinoma cells	Non-malignant urinary papillary urothelial tumor cells	TNTs	Lu et al., 2017
Human MSCs	Human CMs	n.s.	Mahrouf-Yorgov et al., 2017
Human MSCs	Human ECs	n.s.	Mahrouf-Yorgov et al., 2017
Human CMs	Human MSCs	n.s.	Mahrouf-Yorgov et al., 2017
Human ECs	Human MSCs	n.s.	Mahrouf-Yorgov et al., 2017
BM stromal cells*	Acute myeloid leukemia cells*	TNTs	Marlein et al., 2017
Mesenchymal stromal cells*	Macrophages*	EVs	Morrison et al., 2017
Human healthy astrocytes	Human stressed astrocytes	TNTs	Rostami et al., 2017
Human multipotent MSCs	Rat astrocytes	TNTs	Babenko et al., 2018
Human multipotent MSCs	Neuron-like PC12 pheochromocytoma ρ0 cells	TNTs	Babenko et al., 2018
MSCs	Neural stem cells	Actin-based intercellular structures	Boukelmoune et al., 2018
Monkey kidney cells	Monkey kidney cells	TNTs	Guo et al., 2018
Porcine alveolar macrophages	Porcine alveolar macrophages	TNTs	Guo et al., 2018
Porcine umbilical cord MSCs	Porcine alveolar macrophages	TNTs	Guo et al., 2018
Endothelial progenitor cells	Brain ECs	Endothelial progenitor cell–derived mitochondrial particles	Hayakawa et al., 2018
Myeloid-derived regulatory cells	T Cells	ECVs	Hough et al., 2018
Cardiac myofibroblasts	Cardiomyocytes	Nanotubes	Shen et al., 2018
T cell acute lymphoblastic leukemia cells	MSCs	TNTs	Wang e al., 2018
MSCs	T Cell acute lymphoblastic leukemia cells	TNTs	Wang et al., 2018
Human induced pluripotent stem cell–derived MSCs	Human bronchial epithelium cells	TNTs (Cx43 mediated)	Yao et al., 2018
Human induced pluripotent stem cell–derived MSCs*	Murine epithelial cells*	TNTs (Cx43 mediated)	Yao et al., 2018
Scattered tubular-like cells	Tubular epithelial cells	ECVs	Zou et al., 2018
MSCs	Acute lymphoblastic leukemia cells	TNTs	Burt et al., 2019
BM MSCs	Human ECs	TNTs	Feng et al., 2019
Human astrocytes	Human astrocytes	n.s.	Gao et al., 2019
Human neuronal cells	Human astrocytes	n.s.	Gao et al., 2019
Cancer-associated fibroblasts	Prostate cancer cells	Cellular bridges	Ippolito et al., 2019
Cancer-associated fibroblasts*	Prostate cancer cells*	Cellular bridges	Ippolito et al., 2019
BM-derived MSCs	Proximal tubular epithelial cells	n.s.	Konari et al., 2019
BM-derived MSCs*	Proximal tubular epithelial cells*	n.s.	Konari et al., 2019
Rat MSCs	Rat neurons	Gap junction intercellular communication	Li et al., 2019
Rat MSCs*	Rat neurons*	Gap junction intercellular communication	Li et al., 2019
Astrocytes	Primary rat neuronal cells	n.s.	Lippert and Borlongan, 2019
BM stromal cells	MM cells	TNTs	Marlein et al., 2019
BM stromal cells	Hematopoietic stem cells	Gap junction	Mistry et al., 2019
BM stromal cells*	Hematopoietic stem cells*	Gap junction	Mistry et al., 2019
Astrocytes	Neurons	n.s.	English et al., 2020
Murine hematopoietic stem and progenitor cells*	Murine mesenchymal stromal cells*	Cell-contact dependent, Cx43-mediated	Golan et al., 2020
Murine CMs*	Murine macrophages*	Cardiomyocyte-derived exophers (subcellular particles)	Nocolas-Avila et al., 2020
Mesenchymal stromal cells	Islet β cells	TNTs	Rackham et al., 2020
Murine white adipocytes*	Murine macrophages*	n.s.	Brestoff et al., 2021
Human MSCs	Injured alveolar epithelial cells	Cx43-containing gap junctional channels	Huang et al., 2021
Platelets	MSCs	Dynamin-dependent clathrin-mediated endocytosis	Levoux et al., 2021
Platelets*	MSCs*	Dynamin-dependent clathrin-mediated endocytosis	Levoux et al., 2021
BM stromal cells	Myeloma cells	TNTs and partial cell fusion	Matula et al., 2021
Myeloma cells	BM stromal cells	TNTs and partial cell fusion	Matula et al., 2021
Murine ovarian follicles	Murine ovarian follicles	Gap junction internalization	Norris, 2021
Glioblastoma stem cells	Glioblastoma stem cells	TNTs	Pinto et al., 2021
3D-glioblastoma organoids	3D-glioblastoma organoids	TNTs	Pinto et al., 2021
Human breast epithelial cancer cells	Human breast epithelial cancer cells	ECVs	Rabas et al., 2021
Murine high-metastatic lung carcinoma cells	Murine low-metastatic lung carcinoma cells	ECVs	Takenaga et al., 2021
Murine low-metastatic lung carcinoma cells	Murine high-metastatic lung carcinoma cells	ECVs	Takenaga et al., 2021
Murine high-metastatic lung carcinoma cells*	Murine low-metastatic lung carcinoma cells and cancer-associated fibroblasts*	ECVs	Takenaga et al., 2021
MSCs	Neurons	Cell-to-cell contact	Tseng et al., 2021
Human glioblastoma cells	Human primary astrocytes	TNTs	Valdebenito et al., 2021
Human neurons	Human astrocytes	TNTs	Lampinen et al., 2022
Murine neurons	Murine astrocytes	TNTs	Lampinen et al., 2022
Murine brown adipocytes*	Murine adipocytes*	ECVs	Rosina et al., 2022
Effector immune cells	Breast cancer cells	TNTs	Saha et al., 2022
T cells*	Lung carcinoma cells*	TNTs	Saha et al., 2022
T cells*	Melanoma cells*	TNTs	Saha et al., 2022

* in vivo work

Note: TNTs, nanotubes, protrusions, microtubes, actin-based intercellular structures, cellular bridges—types of filamentous tubes that function as intercellular bridges. n.s., not specified in original paper.

In vivo relevance of HMT has been established by several key publications. HMT from MSCs grafted into lungs of mice challenged with lipopolysaccharide (LPS) was documented using mitochondria-competent MSCs, which alleviated the injury (Islam et al., 2012). Using a similar system, a subsequent paper reported on a link of the therapeutic effect of grafted MSCs with the Miro-1 protein (Ahmad et al., 2014), indicating involvement of the kinesin mobility system (MacAskill and Kittler, 2010). Tan and colleagues demonstrated that horizontal transfer of mitochondria into respiration-deficient cancer cells restored their tumorigenic potential and allowed tumor formation (Tan et al., 2015). To date, several other studies demonstrate that HMT occurs in vivo from MSCs to recipient cells to protect the latter or to repair their mitochondrial injury (Ippolito et al., 2019; Crewe et al., 2021; Moschoi et al., 2016; Jackson et al., 2016a; Jackson et al., 2016b), as well as in a variety of other systems with functional consequences. This is consistent with respiratory competent mitochondria being essential for cancer (Wallace, 2012).

## Pathophysiological and phylogenic evidence of HMT and its role in cancer

Mitochondrial dysfunction is associated with many types of human disease (Paliwal et al., 2018; Liu et al., 2014; Berridge et al., 2020). Transfer of healthy mitochondria appears to be an effective rejuvenation process in several types of damaged cells, such as cancer cells, epithelial cells, ECs, and CMs (Paliwal et al., 2018), in the context of multiple diseases. These include ischemia-reperfusion models (Liu et al., 2014), amelioration of acute renal ischemia/reperfusion injury (Gu et al., 2016a), recovery of mitochondrial function in rat CMs after ischemia/reperfusion injury (Han et al., 2016), attenuation of alveolar destruction and altered severity of fibrosis in models of cigarette smoke–induced lung damage (Li et al., 2014), neuroprotective effects, and decline of infarct volume in the brain (Babenko et al., 2015). Moschoi and colleagues found that acute myeloid leukemia cells take up functional mitochondria from murine or human bone marrow (BM) stromal cells following chemotherapy (Moschoi et al., 2016). Concerning physiology, Lei and Spradling showed that germ cells in primordial germ cysts donate organelles, including mitochondria, to facilitate differentiation of mature mouse oocytes (Lei and Spradling, 2016).

Random accumulation of mtDNA deletions and the subsequent mosaic of respiratory chain deficiencies accelerate the aging process (Baris et al., 2015; Krishnan et al., 2008), and the main purpose of HMT is to restore the respiratory activity of acceptor cells with aberrant mitochondrial function (Patananan et al., 2016). Initial studies that demonstrated intercellular HMT were performed in vitro. Human lung carcinoma cells were depleted of mtDNA, with the resulting ρ^0^ cells devoid of respiratory function. Co-culture with MSCs or skin fibroblasts rescued ρ^0^ cells by import of functional mitochondria (Spees et al., 2006; Katrangi et al., 2007). Human osteosarcoma 143B ρ^0^ cells featured collapse of their mitochondrial electron transfer chain (ETC) function and thymidine kinase activity, with impaired de novo synthesis of pyrimidines (King and Attardi, 1989; Cho et al., 2012). When co-cultured with MSCs, they acquired functional mitochondria. The authors reasoned that HMT likely occurs under situations of highly compromised mitochondrial respiratory function (Cho et al., 2012).

**Figure 1.**
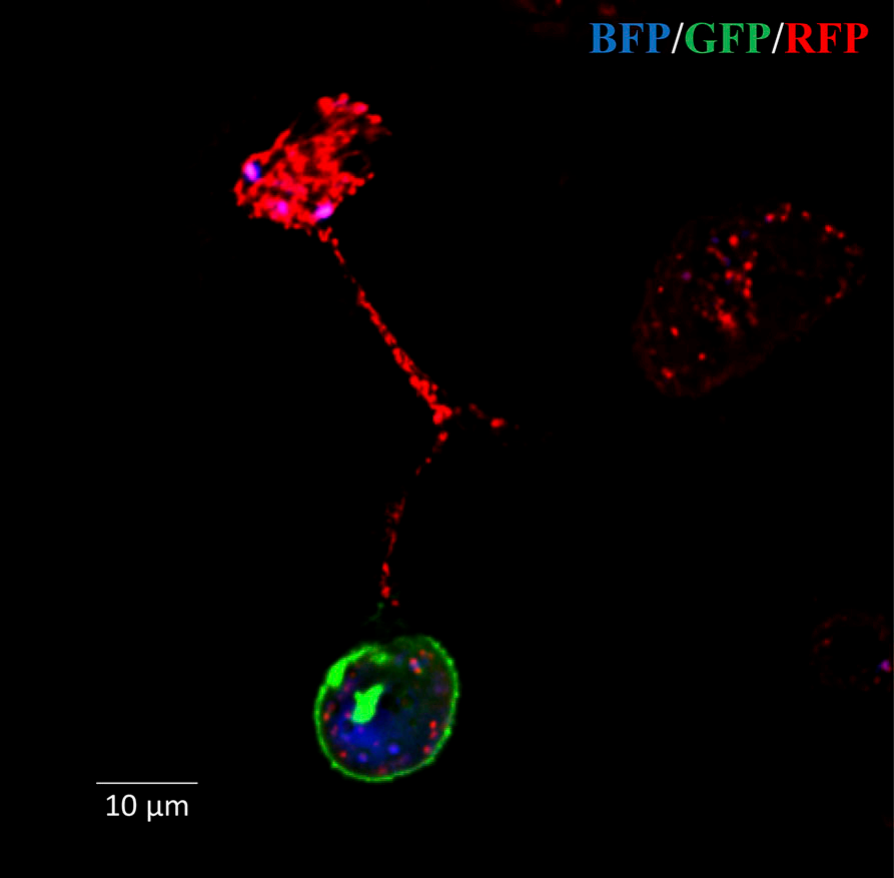
**Movement of mitochondria between MSCs and B16 ρ**^**0**^ **melanoma cells.** MSCs isolated from a transgenic Su9DsRed mouse expressing RFP in their mitochondria were co-cultured with B16 ρ^0^ cells labeled with BFP targeted to nuclei and GFP targeted to the plasma membrane. Confocal microscopy shows the presence of RFP mitochondria in a TNT connecting an MSC and a B16 ρ^0^ cell (Dong et al., 2017).

We used a similar system to investigate HMT in vivo and found that ρ^0^ tumor cells lacking mitochondrial respiratory function showed delayed tumor growth. Acquisition of mtDNA from host cells in the tumor stroma by ρ^0^ tumor cells re-established respiration as well as tumor-initiating and metastatic potential (Tan et al., 2015). We reported that whole mitochondria were transferred from stromal to ρ^0^ tumor cells with their mtDNA payload prior to recovery of tumorigenic capacity (Dong et al., 2017; Bajzikova et al., 2019; Fig. 1).

While complete mtDNA removal is an extreme case that informs on mechanism but is not likely to occur in cancer, HMT was also reported in a more “natural” cancer settings. BM stromal cells donate mitochondria to acute myeloid leukemic cells in xenotransplants in vivo, conferring resistance to chemotherapy and survival advantage (Moschoi et al., 2016) to support their high energy demand and protection from metabolic stress (Marlein et al., 2017; Wang et al., 2018), as also shown in a mouse model of radiation-exposed glioblastoma multiformae (GBM; Osswald et al., 2015; Saha et al., 2022).

HMT has an intriguing role in transmissible cancer in feral dogs and likely in the Tasmanian devil. For feral dogs, CTVT is transmitted via coitus while Tasmanian devil spreads cancer via saliva entering open wounds. These animals live in partially inbred family packs, lowering the barrier against transfer of genes between individuals. Transmissible cancers in dogs were first described more than a decade ago (Rebbeck et al., 2009). It was then hypothesized that mtDNA damage during cancer progression accumulates to the point of irreversible damage to mitochondrial respiration triggering transfer of healthy mitochondria from donor to cancer cells (Rebbeck et al., 2011). While these two types of cancer are evolutionarily “old,” having developed thousands of years ago (Murchison et al., 2010; Murchison et al., 2012; Murchison et al., 2014; Strakova and Murchison, 2015), little is known about HMT in facial tumors (Kwon et al., 2020), while HMT in CTVT has been well defined with interesting caveats (Strakova et al., 2016; Strakova et al., 2020). Sequencing nDNA and mtDNA from CTVT tumors across ∼450 individual animals from countries around the world showed no homology in nDNA, while there were only five haplotypes, indicating at least five transfers of mitochondria with mtDNA over 1,500 yr. Interestingly, clade 1 haplotype bearers were found in countries as distal as Australia, India, Romania, Tanzania, and Chile, and clade 5 haplotype bearers were found only in India. Further work by this group (Strakova et al., 2020) showed that HMT in CTVT results in positive selection of the transferred mtDNA.

## Mitochondrial transfer in non-cancerous systems

The notion that HMT between cells is a more general phenomenon that occurs also in normal physiology is now emerging, and HMT plays a role not only in the context of cancer and other pathologies but also under physiological conditions (Lei and Sprandling, 2016; Jayaprakashet al., 2017; Torralba et al., 2016; Valenti et al., 2021). This is supported by reports of HMT modulating in vivo adipose tissue and heart homeostasis or thermogenesis (Nicolas-Avila et al., 2020; Brestoff et al., 2021; Rosina et al., 2022).

### Mitochondrial transfer in respiratory system injury and inflammation

Tissue injury and inflammation that involve considerable cellular stress may drive HMT. A decade ago, HMT from BM-derived stromal cells to pulmonary alveoli was reported, indicating that transfer of intact mitochondria can contribute to tissue repair in vivo, implying that HMT can be utilized as a beneficial approach in MSC-based therapy (Islam et al., 2012), enhancing cellular bioenergetics with ensuing improved organ function (Sinha et al., 2016). An in vivo study showed that increased expression of Miro1 in MSCs increased HMT from the stem cells into injured bronchial epithelial cells through intercellular TNTs. This resulted in attenuation of epithelial cell apoptosis, reduced infiltration of inflammatory cells, and lower collagen deposition and mucus hypersecretion in the lungs (Ahmad et al., 2014).

Further research confirmed that exposure of the respiratory system to injury or inflammation triggers HMT from MSCs to lung epithelium, using a mouse model of cigarette smoke–induced lung damage (Li et al., 2014). Lung alveolar macrophages were also shown to acquire mitochondria from MSCs by means of TNT-like structures, resulting in enhanced phagocytic activity in a model of pneumonia. This presents a novel mechanism for the anti-microbial effect of MSCs in the acute respiratory distress syndrome (Jackson et al., 2016a; Jackson et al., 2016b).

### Mitochondrial transfer in the cardiovascular system

The cardiovascular system includes cell types with both high and low dependence on mitochondrial ATP production. The former cell type is represented by CMs, the latter by ECs. While generating little ATP in their mitochondria (mtATP), ECs use mitochondrial respiration to support stress resistance and biosynthesis (Magalhaes-Novais et al., 2022; Diebold et al., 2019). Despite these differences, both CMs and ECs engage HMT, suggesting that HMT is not restricted to mtATP-dependent cell types, and emphasizing the role of HMT in stress resistance. An earlier report indicated that mitochondria could move spontaneously from CMs to ECs via transient nanotube-like structures (Koyanagi et al., 2005). Mitochondria were then shown to be endocytosed by CMs and other cells in both in vitro and in vivo models of cardiomyopathy and ischemia, indicating therapeutic potential (Masuzawa et al., 2013; Cowan et al., 2016). MSCs were found to donate mitochondria, rescuing ischemia-exposed CMs and ECs from cell death (Cselenyak et al., 2010; Mahrouf-Yorgov et al., 2017).

Unidirectional mitochondrial transfer was found in the direction of CMs upon co-culture of human MSCs with rat CMs (Boukelmoune et al., 2018), or from myofibroblasts to damaged CMs to attenuate their apoptosis during hypoxia/reoxygenation (Shen et al., 2018). Mitochondria from endothelial progenitor cells can be delivered into damaged brain ECs (Hayakawa et al., 2018). Bidirectional movement of mitochondria between cardiofibroblasts and CMs (He et al., 2011), or between MSCs and CMs or ECs (Mahrouf-Yorgov et al., 2017) was reported. Mitochondrial dysfunction plays a central pathogenic role in neonatal cardiomyopathy, and HMT improves CM bioenergetics and viability in rats exposed to pre-gestational diabetes (Louwagie et al., 2021).

Recently, it was shown that cardiac tissue releases “damaged” mitochondria via extracellular vesicles (ECVs) called exospheres are taken up by macrophages via their Mertk surface receptors and disposed of (Melentijevic et al., 2017), contributing to mitochondrial homeostasis in the heart (Nicolas-Avila et al., 2020). Inter-organ HMT from adipose tissue to CMs, causing “metabolic pre-conditioning” of the heart (Crewe et al., 2021), has also been demonstrated.

### Mitochondrial transfer in the nervous system

The central nervous system (CNS) controls basic physiological functions, emotional changes, and mental health (Zheng et al., 2019). Mitochondria serve not only as the “powerhouse” of neurons but also play essential roles in metabolizing neurotransmitters, buffering Ca^2+^, and sending signals modulating neuronal survival (Course and Wang, 2016; Misgeld and Schwarz, 2017). Vesicles containing mitochondria are transported unidirectionally between neuronal cells (Rustom et al., 2004). Contrary to our understanding that healthy cells degrade their own mitochondria, the organelles move from retinal ganglion axons to adjacent astrocytes in the optic nerve to be disposed of via trans-mitophagy (Davis et al., 2014), and neural trans-mitophagy was shown in a mouse model of Alzheimer’s disease (AD; Lampinen et al., 2022). In the hippocampus, motility of axonal mitochondria affects the pre-synaptic strength (Sun et al., 2013). Intercellular mitochondrial transport is critical in maintaining the healthy state of mitochondria in axons and homeostasis of the CNS (Nguyen et al., 2014), and this process plays an important role in various neurological and psychiatric disorders (Zheng et al., 2019; Picard et al., 2015; Gaetani et al., 2022).

Mitochondrial motility not only impacts on tissues/cells in the CNS but also in the peripheral nervous tissue. A recent experiment demonstrated that exogenous mitochondria transplanted into injured rat spinal cord contribute to acute maintenance of bioenergetics as well as functional recovery after spinal cord injury (Gollihue and Rabchevsky, 2017). Mitochondria can move from BM MSCs to oxygen and glucose-deprived neurons, improving their survival, decreasing neuronal apoptosis, and promoting locomotor function recovery in rats after spinal cord injury, indicating potential stem cell therapy (Li et al., 2019), while mitochondria from multipotent MSCs move to neurons or astrocytes, leading to restoration of respiration in recipient cells and alleviation of ischemic damage (Babenko et al., 2015; Babenko et al., 2018).

Astrocytes have been suggested as potential mitochondrial donors. An in vivo mouse model of stroke indicated that astrocytes release functional mitochondria that were delivered to damaged neurons, resulting in ischemic injury repair and neuronal recovery (Hayakawa et al., 2016; Berridge et al., 2016)*.* Mitochondria from astrocytes transfer to cerebrospinal fluid after subarachnoid hemorrhage, both in a rat model and humans (Chou et al., 2017), and stressed astrocytes acquire functional mitochondria from healthy astrocytes via direct contact or TNTs facilitating their own recovery (Rostami et al., 2017). Astrocytes transfer healthy mitochondria to neurons after cisplatin treatment to restore “neuronal health” (English et al., 2020). Mitochondria were found to move between astrocytes and neurons by a process involving CD38/cADP-ribose signaling and mitochondrial Rho GTPases (Miro1, Miro2; Hayakawa et al., 2016; Gao et al., 2019). Mitochondrial transfer (or exogenous delivery) may open an avenue for treatment of neurological diseases such as stroke and spinal cord injury (Han et al., 2020).

### Mitochondrial transfer in other systems

Mitochondria collected from murine hepatocytes improve embryonic development following transfer to fertilized murine zygotes from young and older mice (Yi et al., 2007). Cells in primordial oocyte cysts transfer mitochondria to definitive oocytes (Lei and Spradling, 2016). Also, donation of mitochondria by MSCs protects retinal ganglion cells against mitochondrial CI defect-induced degeneration (Jiang et al., 2019).

Mitochondria released from damaged somatic cells (CMs or ECs) can be engulfed by MSCs and rapidly degraded. As a consequence, elevation of heme levels in the cytosol of recipient MSCs triggered upregulation of heme oxygenase-1, a stress-inducible protein endowed with cytoprotective properties that converts toxic heme into health-promoting compounds (Gozzelino et al., 2010). Thus, heme oxygenase-1 activation increased mitochondrial biogenesis and protected against somatic cell apoptosis by stimulating HMT from MSCs to damaged cells (Mahrouf-Yorgov et al., 2017). Although the majority of studies on HMT focus on stress-linked reactions, it is of importance to investigate the potential role of HMT in maintaining tissue homeostasis (Liu et al., 2021).

In vitro studies found that intercellular transfer of mitochondria can be bidirectional. This is exemplified by exchange of mitochondria between renal tubular cells and mesenchymal multipotent stromal cells (Plotnikov et al., 2010). Bidirectional exchange of mitochondria was detected under normal culture conditions between human vascular smooth muscle cells and BM MSCs, and this process promoted MSC proliferation (Vallabhaneni et al., 2012). Human BM-derived MSCs transfer mitochondria to macrophages in vivo via TNTs, resulting in enhanced macrophage phagocytosis, a novel mechanism promoting anti-microbial function of MSCs (Jackson et al., 2016a; Jackson et al., 2016b). A report documented HMT from MSCs to corneal epithelial cells to protect them from mitochondrial damage (Jiang et al., 2016). In retinal cells, mitochondria and endocytic organelles were found inside TNTs, indicating that mitochondria may be transferred between individual cells or between retinal pigment epithelium cells and photoreceptors (Wittig et al., 2012). Mitochondria move from adipose tissue to macrophages in order to maintain white adipose tissue homeostasis, with positive impact on obesity (Brestoff et al., 2021). Consistent with this, damaged mitochondria transfer from brown adipocytes to macrophages to regulate thermogenesis (Rosina et al., 2022). Finally, several reports indicate that platelets donate mitochondria to neutrophils or MSCs, eliciting immune (Boudreau et al., 2014) or regenerative responses (Levoux et al., 2021).

Pathological and non-pathological systems in which mitochondrial transfer has been documented are summarized in Table 2.

**Table 2.** Pathological and non-pathological systems featuring mitochondrial transfer

Condition		Result	Reference
Pathological	Lung carcinoma	Rescued mitochondrial function	Spees et al., 2006
	Osteosarcoma	Rescued mitochondrial function	Cho et al., 2012
	Acute lung injury	Cellular protection and tissue repair	Islam et al., 2012
	Ischemia	Preserving myocardial energetics, cell viability, and enhanced post-infarct cardiac function—protect the heart from ischemia-reperfusion injury	Masuzawa et al., 2013
	Allergic airway inflammation	Enhanced rescue of epithelial injury	Ahmad et al., 2014
	Chronic obstructive pulmonary disease	Attenuation of cigarette smoke–induced lung damage	Li et al., 2014
	Ischemia	Cardioprotection from ischemia-reperfusion injury	Cowan et al., 2016
	Cerebral ischemia	Amplified cell survival signals—neurorecovery	Hayakawa et al., 2016
	Acute respiratory distress syndrome	Enhancement of phagocytic activity of lung alveolar macrophages	Jackson et al., 2016a; Jackson et al., 2016b
	Acute myeloid leukemia	Resistance to chemotherapy	Moschoi et al., 2016
	Canine transmissible venereal tumor	Acquisition of functional mtDNA	Strakova et al., 2016, 2020
	Bladder cancer	Increased invasiveness	Lu et al., 20178
	Acute respiratory distress syndrome	Anti-inflammatory and highly phagocytic macrophage phenotype resulting in amelioration of lung injury	Morrison et al., 2017
	PD	Acquisition of functional mitochondria	Rostami et al., 2017
	Oxygen-glucose deprivation	Restoring brain endothelial energetics and barrier integrity	Hayakawa et al., 2018
	Hypoxia/reoxygenation injury	Attenuation of CM apoptosis	Shen et al., 2018
	Asthma	Alleviated asthmatic inflammation	Yao et al., 2018
	Diabetic nephropathy	Structural and functional restoration of renal proximal tubular epithelial cells	Konari et al., 2019
	MM	Enhanced mitochondrial metabolism, protumoral effect	Marlein et al., 2019
	Neonatal cardiomyopathy	Improvement of CM bioenergetics and viability in male rats exposed to pre-gestational diabetes	Louwagie et al., 2021
	Lung carcinoma	Enhancement of metastatic potential during tumor progression	Takenaga et al., 2021
	Cerebral ischemia	Increased neuronal survival and improved metabolism	Tseng et al., 2021
	Glioblastoma	Adaptation of non-tumor astrocytes to tumor-like metabolism and hypoxia conditions	Valdebenito et al., 2021
	AD	Increased transmitophagy of defective neuronal mitochondrial, potential alleviation of AD pathology and symptoms	Lampinen et al., 2022
Non-pathological	Cardiac homeostasis	Preserved metabolic stability and organ function	Nicolas-Avila et al., 2020
	White adipose tissue homeostasis	Metabolic homeostasis, impairment leads to obesity	Brestoff et al., 2021
	Metabolic preconditioning of the heart	Cardio-protection against lipotoxic or ischemic stresses elicited by obesity	Crewe et al., 2021
	Wound healing	Promotion of pro-angiogenic activity of MSCs via their metabolic remodeling	Levoux et al., 2021

## Modes of HMT

**Figure 2.**
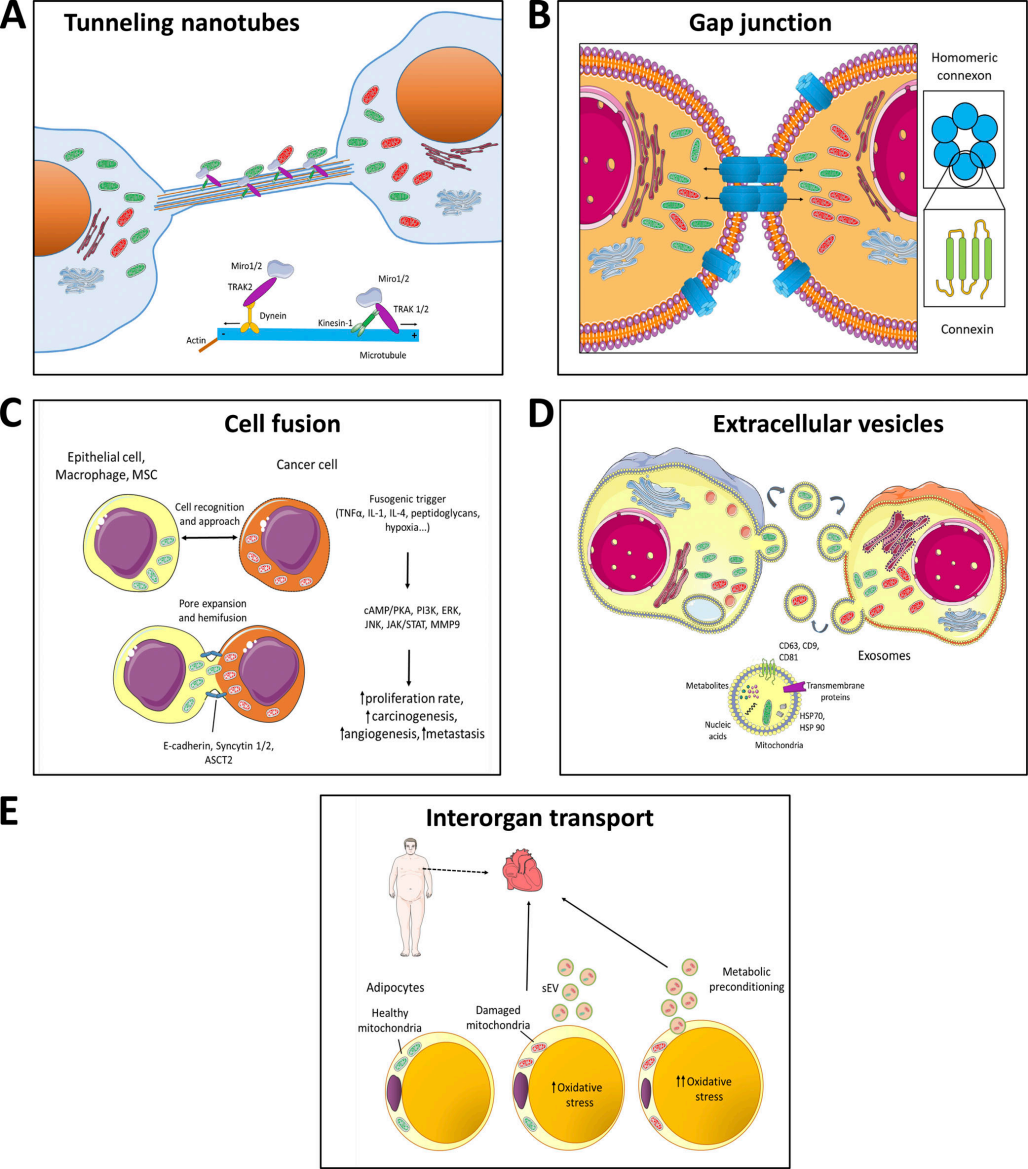
**Models of horizontal transfer of mitochondria. (A)** Horizontal transfer of mitochondria by TNTs. TNTs are in general formed by F-actin filaments. In case of intercellular transfer of mitochondria, TNTs also contain microtubules and are thicker, thus able to transport bulkier structures. Transport of mitochondria along these cytoskeletal elements is propelled by dynein and kinesin motor complexes consisting of several adaptor proteins, such as Miro1 or Miro2, that are integrated in the outer mitochondrial membrane and facilitate mitochondrial transport not only along microtubules but also along actin filaments (together with Myo19). Formation of TNTs starts either as an actin-driven protrusion of the cell membranes of the two cells involved or as the dislodgement of two previously attached cells that during the partition from each other form the TNT. **(B)** Horizontal transfer of mitochondria by gap junctions. Gap junctions were shown to involve connexin structures, i.e., protein complexes consisting of six subunits of connexin proteins, such as Cx43. Two juxtapositioned connexon channels form pores connecting two neighboring cells, allowing for bidirectional transport of ions, signaling molecules or whole mitochondria. **(C)** Horizontal transfer of mitochondria by cell fusion. Cell fusion is a process that is relatively common in cancer progression and comprises several steps. At first two cells are recognized via the so-called fusogenic trigger that could involve different signaling molecules depending on the cell type (TNFα, IL-1, IL-4, and others) or specific conditions (e.g., hypoxia). The cells then approach each other and, using several cell–cell adhesion molecules, such as E-cadherin, syncytin-1 and -2, or ASCT2, which form pore expansions, “fusion” of the two neighboring cells occurs. This yields a cell that shares mitochondria from the two original cells. During this process, several signaling pathways are triggered that result in higher tumorigenesis and increased metastatic potential of the cancer cells. **(D)** Horizontal transfer of mitochondria by ECVs. Transfer of mitochondria via ECVs involves small double membrane structures that are formed by blebbing of plasma membrane. In contrast, exosomes are of endosomal origin and can transport various cargo including signaling molecules (different metabolites), trans-membrane proteins, nucleic acids, amino acids, and organelle fragments. **(E)** Inter-organ transport of mitochondria. This mode of mitochondrial horizontal transfer has been recently shown for the organelles moving from adipocytes to the heart tissue. In this particular case, damaged mitochondria from energetically stressed adipocytes of obese patients are transferred via small extracellular vesicles (sEV) to the blood circulation and are taken up by CMs of the heart tissue, triggering small ROS burst. This process results in compensatory antioxidant signaling in the heart muscle, causing metabolic pre-conditioning.

Intercellular mitochondrial transfer is important in multiple scenarios during physiology and pathophysiology. Mechanisms of HMT between cells are diverse as discussed in more detail below, including tunnelling nanotubes/cytoplasmic bridges, gap junctions, cell fusion, endocytosis of vesicles, or as free organelles (Moschoi et al., 2016; Osswald et al., 2015; Masuzawa et al., 2013; Cowan et al., 2016; Hayakawa et al., 2016; Wang and Gerdes, 2015; Herst et al., 2018). The various modes of HMT are depicted schematically in Fig. 2.

### Mitochondrial transfer via TNTs

The most studied mechanism of HMT is by means of F-actin–based cytoplasmic bridges referred to as TNTs (Rustom et al., 2004; Gerdes and Carvalho, 2008; Gurke et al., 2008; Rustom, 2016; Zurzolo, 2021). TNTs are dynamic structures with a diameter of 50–200 nm and length up to several cell diameters. They are derived from plasma membrane and can form within minutes. These cell–cell connections mediate continuity between the cytoplasm of adjacent or remote cells, allowing for trafficking of mitochondria and other organelles, i.e., vesicles, individual molecules (nucleic acids), ions, and even pathogens travelling from donor to recipient cells (Rustom et al., 2004; Sowinski et al., 2008; Bukoreshtliev et al., 2009; Eugenin et al., 2009; Thayanithy et al., 2014; Roehlecke and Schmidt, 2020; Haimovich et al., 2021). Drugs that depolymerize F-actin suppress formation of TNTs (Bukoreshtliev et al., 2009). In several cases microtubules and cytokeratin filaments are present in these structures together with F-actin, their key component (Veranic et al., 2008; Wang et al., 2010).

Two basic mechanisms of TNT formation exist: actin-driven protrusion of the cell membrane that fuses with another cell or its protrusion and “cell dislodgement,” where two initially attached cells part from each other, forming a TNT containing F-actin filaments (Rustom et al., 2004; Ljubojevic et al., 2021). Existence of two different types of TNTs, with or without microtubules, implies different functions. TNTs without microtubules likely serve more as a short-distance connection, while those containing microtubules and with a wider bore serve for transport of molecules and organelles over longer distances (MacAskill and Kittler, 2010; Zampieri et al., 2021).

Mitochondria associate with actin or tubulin fibers by means of Miro proteins, playing a role in mitochondrial transport (Nahacka et al., 2021). In vitro experiments show that overexpression of Miro-1 in MSCs increased the metabolic/bioenergetic benefit of HMT following oxidative damage of recipient cells (Ahmad et al., 2014; Tseng et al., 2021). We have shown a role for TRAK-1, another adaptor protein linking mitochondria to motor proteins like kinesin (KIF5A), enabling movement of mitochondria at long range and crossing “crowded” environments (Henrichs et al., 2020). An interesting phenomenon is bidirectional transfer of mitochondria shown for several systems (see above) and as can be expected for cancer. One can envisage that mitochondria moving to the plus ends of tubulin fibers use the kinesin mobility system, while those moving in the anterograde direction use dynein as the motor protein. That these two mitochondrial transport systems do not collide can be due to a mechanism analogous to the movement of interflagellar trains in cilia (Stepanek and Pigino, 2016; Jordan et al., 2018; Pigino, 2021).

Occurrence of TNTs is a rather infrequent process taking place under a wide range of physiological conditions (development, regeneration, cell migration, etc.) and different pathological situations including tumor formation and metastasis (Lou et al., 2012; Ariazi et al., 2017; Valdebenito et al., 2021; Takenaga et al., 2021). TNTs present a component of the tumor microenvironment and can form in solid tumors and in primary cancer cells, playing an important role in cancer cell pathogenesis and invasion (Lou et al., 2012). Spontaneously formed TNTs mediate hetero-cellular exchange between various cancer and stromal cells for transfer of mitochondria, promoting tumor progression. To maintain HMT, TNTs form as previously shown in a model of ischemic vascular disease for injured cells via the so-called “find me” signals, such that phosphatidylserine exposed on the surface of ECs facilitates their connection with neighboring MSCs (Liu et al., 2014).

Stress signals generated during cellular mitochondrial damage, together with elevated levels of ROS, prompt donor cells to enhance their bioenergetics and initiate mitochondrial donation to injured recipient cells (Paliwal et al., 2018; Mahrouf-Yorgov et al., 2017; Zhang et al., 2010). Formation of TNTs via actin-driven protrusions of plasma membrane in MSCs is initiated by the cytokine TNF-α, regulating the TNFα/NF-κB/TNF-αip2 signaling pathway that leads to F-actin polymerization and TNT formation (Zhang et al., 2010; Hase et al., 2009). In 2006, Spees and colleagues reported that healthy mitochondria move from MSCs to cells with dysfunctional mitochondria, restoring their respiration (Spees et al., 2006). In a model of acute respiratory distress syndrome, TNTs were found to form between MSCs and lung macrophages, moving mitochondria across, resulting in higher phagocytic activity of lung macrophages (Jackson et al., 2016a; Jackson et al., 2016b). In a model of simulated ischemia/reperfusion injury, mitochondrial transfer occurred from MSCs to injured H9c2 cardiomyoblasts via TNTs, rescuing cardiac cells from apoptosis (Han et al., 2016). Mitochondria move in a bidirectional manner between MSCs and vascular smooth muscle cells via TNTs to initiate their proliferation (Vallabhaneni et al., 2012). BM MSCs move mitochondria to myeloma cells via TNTs, initiated by CD38 expression on myeloma cells (Marlein et al., 2019). A study of ECs exposed to chemotherapeutic stress showed mitochondrial transfer from BM MSCs via TNTs (Feng et al., 2019).

Donor cells other than MSCs also transfer mitochondria via TNTs to recipient cells. Within the same cell type, healthy cells donate mitochondria to their injured counterparts. Pheochromocytoma (PC12) cells exposed to UV light were rescued when co-cultured with untreated PC12 cells. This was promoted by TNTs formed by the stressed cells, allowing for transfer of functional mitochondria and rescuing the cells from apoptosis (Wang and Gerdes, 2015). Porcine reproductive and respiratory syndrome virus-infected cells were rescued from apoptosis/necrosis in early stages of infection by transfer of functional mitochondria via TNTs from uninfected cells (Guo et al., 2018).

TNTs also form between lung epithelial cells over distances from 1 μm to more than 100 μm (Kumar et al., 2017). In vitro and in vivo evidence shows that TNTs promote intercellular HMT between heterogeneous cancer cells, followed by increased invasiveness of bladder cancer cells (Lu et al., 2017). Pathologically stressed astrocytes with swollen ER and impaired mitochondrial dynamics signal to healthy astrocytes, which promotes HMT via TNTs (Rostami et al., 2017). A recent study showed mitochondrial transfer from astrocytes to GBM cells via TNTs as an adaptation to hypoxic and nutrient deficiencies in the tumor microenvironment (Valdebenito et al., 2021). TNTs were also found to form in retinal pigment epithelium, with HMT maintaining homeostasis of the tissue (Wittig et al., 2012; Chinnery and Keller, 2020), and in cancer spheroid 3D structures, in which cancer and stromal cells communicate via TNTs with ensuing HMT from ECs to cancer cells, contributing to chemotherapy resistance (Pasquier et al., 2013).

### Mitochondrial transfer via gap junctions

Gap junctions couple nearly all cells that line external and internal surfaces of the human body, being known for transferring small molecules between cells (Goodenough and Paul, 2009). During gap junction internalization, one cell engulfs small parts of the neighboring cell (Bettadapur et al., 2020). The integral gap junction protein, connexin 43 (Cx43), is involved in ischemia/reperfusion damage of myocardial and cerebral tissue, implying HMT (Islam et al., 2012; Antanaviciute et al., 2014; Schulz et al., 2015; Norris, 2021; Qin et al., 2021; Rodriguez-Sinovas et al., 2021). Gap junction associated HMT involves Cx43 gap junctions within ovarian follicles (Norris, 2021). Mitochondrial transfer from donor Cx43-expressing hematopoietic progenitors to stromal cells is required to support BM regeneration following irradiation (Golan et al., 2020). Promotion of pro-inflammatory status following bacterial LPS challenge was found to enable injured pulmonary epithelial cells to fuse with human stem cells using Cx43 during the formation of TNTs (Islam et al., 2012; Bagheri et al., 2020). Additionally, HMT via gap junctions occurs from BM MSCs to injured motor neurons to protect them from apoptosis (Li et al., 2019).

Interestingly, the role of Cx43 in the process of TNT formation was also shown in other models of HMT, for example in asthmatic inflammation, virus infection and leukemia (Smith et al., 2011; Okafo et al., 2017; Griessinger et al., 2017), indicating that the connexin system may be involved in mitochondrial transfer other than via gap junctions.

### Mitochondrial transfer via cell fusion

Cell fusion is a process of two individual cells fusing their plasma membranes, sharing organelles and cytosolic compounds while their nuclei remain intact (Bagheri et al., 2020). Cytosolic constituents and organelles are hence evenly or, in some cases, partially shared between juxtaposed cells (Aguilar et al., 2013), resulting in mitochondrial delivery into recipient cells (Gomzikova et al., 2021), being common in myogenesis and placental development. An in vivo study showed that BM-derived cells fuse with neurons, CMs, and hepatocytes, raising the possibility that cell fusion may contribute to cellular homeostasis (Alvarez-Dolado et al., 2003). Stem cells can also fuse with neurons (Cusulin et al., 2012) and hepatocytes (Terada et al., 2002). Cell fusion in target organs can be driven by injury and inflammation (Weimann et al., 2003), irradiation (Alvarez-Dolado et al., 2003), or hypoxia-induced apoptosis (Noubissi et al., 2015). Cells of myeloid and lymphoid lineages fuse with cells in different tissues in response to injury or inflammation (Nygren et al., 2008). Cell fusion also occurs following co-culture of mouse CMs with human multipotent adipose-derived stem cells, resulting in HMT into CMs (Acquistapace et al., 2011).

### Mitochondrial transfer via ECVs

ECVs are lipid-bound structures secreted into the extracellular space (EL Andalousi et al., 2013; Yáñez-Mó et al., 2015; Zaborowski et al., 2015). They include micro-vesicles (MVs) of 0.1–1 μm in diameter, and exosomes with diameter of 30–150 nm (Doyle and Wang, 2019; Isaac et al., 2021). The original notion was that exosomes and MVs are used by cells for disposal of unwanted material to maintain homeostasis (Yáñez-Mó et al., 2015). However, it became evident that ECVs are involved in cell-to-cell communication at a longer range, often provoking changes in the recipient cell (Isaac et al., 2021; White et al., 2006; Harding et al., 2013), also providing energy (Kumar et al., 2021). MVs can encapsulate mitochondria (∼0.5 μm in diameter) and deliver them to target cells (Vignais et al., 2017). Encapsulation of mitochondria into ECVs could be a rescue mechanism to release oxidative stress and to clear depolarized mitochondria (Gomzikova et al., 2021; Phinney et al., 2015), maintaining tissue homeostasis (Brestoff et al., 2021).

Delivery of mitochondria by ECVs participates in immune regulation, modulating the function of macrophages and neutrophils and other cells, including alveolar epithelial cells, neurons, and ECs, and contributing to intercellular communication (She et al., 2021). The first report of ECV-mediated HMT is a study showing HMT from BM MSCs to injured lung alveolar epithelial cells in a model of acute lung injury (Islam et al., 2012). ECV-mediated HMT from MSCs to macrophages, contributing to amelioration of lung injury, enhances the phagocytic capacity of M2-type alveolar macrophages and reduces secretion of TNF-α, suppressing lung inflammation (Morrison et al., 2017). Using a mouse model of focal cerebral ischemia, astrocytes were shown to use ECVs to transfer functional mitochondria to protect neurons from hypoxia and glucose deprivation (Hayakawa et al., 2016).

ECV-mediated HMT was found to occur between renal scattered tubular cells and tubular epithelial cells to alleviate renal stenosis and to recover mitochondrial respiration (Zou et al., 2018). Mitochondria derived from MSCs move to macrophages via ECVs to increase ATP production (Phinney et al., 2015). As a consequence of stimulating neutrophil activation and promoting their pro-inflammatory responses, platelets shed mitochondria in ECVs that are taken up by neutrophils in a damage-related model (Boudreau et al., 2014). Mitochondria encapsulated in ECVs can integrate into T cells to affect their mitotic processes (Hough et al., 2018). We showed that vesicular mitochondria released by platelets are internalized by MSCs, activating their pro-angiogenic properties (Levoux et al., 2021). These findings indicate that delivery of mitochondria by ECVs allows for communication between cells and participation in regulating the immune system and tissue repair processes, applicable for ECV-based mitochondrial therapy (She et al., 2021).

An interesting form of HMT between cells (or even organs) in response to stress involves release of damaged mitochondria from adipocytes in ECVs, with individual mitochondria originating from mitochondria-derived vesicles formed by budding from the mitochondrial network (Crewe et al., 2021). The function of mitochondria-derived vesicles is transport of cargo between mitochondria and other organelles, including ECVs (Todkar et al., 2021). ECVs are taken up by CMs, where they exert elevated oxidative stress that causes “metabolic” pre-conditioning of the heart (Crewe et al., 2021). We have recently shown that mitochondria move from platelets to MSCs in ECVs and as “isolated” mitochondria, ∼50% of each (Levoux et al., 2021). Both in the case of isolated mitochondria and those in ECVs, the organelles are taken up by clathrin-dependent endocytosis. Vesicles released by cancer cells contain “naked” mtDNA, which causes higher invasiveness of recipient cells (Rabas et al., 2021). While some of these reports are indicative of the existence of isolated mitochondria (or mtDNA) within the extracellular compartment, systematic studies on the mechanism by which these organelles are shed by donor cells and internalized by acceptor cells are lacking.

A novel means of HMT has recently emerged involving migrasomes (Ma et al., 2015) that form on retraction fibers of migrating cells via tetraspanin microdomains (Huang et al., 2019). These structures contain damaged mitochondria that are disposed of by the process of mitocytosis (Lu et al., 2020; Jiao et al., 2021). While migrasomes have been suggested to play a role in stroke (Schmidt-Pogoda et al., 2018), they likely deliver mitochondria (albeit damaged) to other cells (Yu and Yu, 2022).

## Consequences of mitochondrial transfer

HMT has been shown to play a critical role in cell and tissue regeneration, and in damage repair, or may contribute to healing processes in brain injury, ischemic heart disease, muscle sepsis, stroke, and lung disorders (Paliwal et al., 2018). Various stress signals act as triggers of HMT, occurring primarily from MSCs to recipient cells, often in response to increased levels of ROS, damaged mitochondria/mtDNA, or inflammation (Zhang et al., 2016; Mistry et al., 2019). Cells injured by oxidative stress due to dysfunctional mitochondria send environmental cues to MSCs, triggering HMT (Islam et al., 2012; Ahmad et al., 2014). Bidirectional transfer of mitochondria occurs between MSCs and the surrounding environment where MSCs dispose of damaged mitochondria. Using rotenone to induce oxidative stress in corneal epithelial cells, HMT was initiated from MSCs, enhancing the survival capacity of the cells, paralleled by elevated mitochondrial respiration and enhanced corneal wound healing (Jiang et al., 2016). In cells derived from a patient with the mitochondrial disease MERRF (myoclonus epilepsy with ragged-red fibers), MSCs donate mitochondria leading to the rescue of injured cells via improved aerobic respiration, suppressed apoptosis and decreased mutational load and oxidative damage (Chuang et al., 2017).

In cancer, chemotherapy is mostly directed at cancer cells, driving them into apoptosis. Tumor cells importing mitochondria from stromal cells are better protected from apoptosis induced by chemotherapy or radiation therapy (Osswald et al., 2015; Pasquier et al., 2013). HMT from BM MSCs to acute myeloid leukemia cells in vivo confers chemoresistance and promotes their survival (Moschoi et al., 2016). Acquisition of new mitochondria via HMT generally increases cell fitness and overall resilience (Bererdi and Fantin, 2011; Suomalainen, 2019). Therefore, an important consequence of HMT is increased stress resistance of recipient cells.

Another consequence of HMT particularly important for rapidly proliferating cancer cells relates to the ETC playing an essential role in anabolic cell proliferation (Vander Heiden et al., 2009; Koppenol et al., 2011; Birsoy et al., 2015; Sullivan et al., 2016; Spinelli and Haigis, 2018). Invasive cancer cells favor mitochondrial respiration and increased ATP formation to meet their energy demands (LeBleu et al., 2014). A subpopulation of quiescent tumor cells that self-renew slowly/infrequently are responsible for tumor relapse (Visvader and Lindeman, 2012), and are addicted to OXPHOS for their survival (Kapoor et al., 2014; Viale et al., 2014).

HMT is triggered by severely affected mitochondrial functions, such as mtDNA deletion or treatment with mitochondrial inhibitors (Cho et al., 2012; Wang and Gerdes, 2015). We showed that cancer cells without mtDNA acquire healthy mitochondria from stromal cells, recovering their respiratory ability and tumorigenic capacity (Tan et al., 2015; Dong et al., 2017). These findings demonstrate that mitochondrial respiration is indispensable for tumor formation and progression. Respiration transfers electrons to oxygen, providing a cellular redox state that allows biosynthesis. CIII/IV maintain redox cycling of coenzyme Q (CoQ; re-oxidizing its reduced form to its oxidized counterpart) needed for de novo pyrimidine synthesis via dihydroorotate dehydrogenase (DHODH; Bajzikova et al., 2019; Boukalova et al., 2020). In addition, CoQ as well as CI activity support biosynthesis of other essential metabolites, including aspartate, that feed into nucleotide synthesis pathways (Birsoy et al., 2015; Guarás et al., 2016; Sullivan et al., 2016; Bajzikova et al., 2019; Martínez-Reyes et al., 2020; Murphy and Chouchani, 2022). Conceivably, HMT plays an important role within the tumor microenvironment by maintaining metabolic homeostasis that favors biomass build up (Tan et al., 2015; Mohammadalipour et al., 2020).

Regulation of mtDNA copy number in tumorigenesis (Dickinson et al., 2013), control of respiratory complex formation and function by translational crosstalk (Couvillion et al., 2016), plus epigenetic mechanisms suggest that maintaining optimum bioenergetic balance is a dynamic and adaptive process. Emerging data indicate that, when needed, this balance is maintained via HMT (Lee et al., 2015; Sun et al., 2018). The question of mtDNA copy number and HMT is intriguing. It is unclear at present what extent of mtDNA damage provokes HMT, and the extent to which damage can be reversed by mtDNA repair. GBM cells form tumors in mice with a delay directly related to mtDNA copy number, and this is linked to animal survival (Dickinson et al., 2013). This may have connotations for translational applications, such as cancer therapy.

Mitochondria are not only the primary powerhouse of cells, but also important regulators of life, death, proliferation, motility, stemness, and other functions (Ohta, 2003). Whole mitochondria with mtDNA are “mobile elements” that move between cells, with a role in cancer development, progression, and treatment (Dong et al., 2017; Ishikawa et al., 2008). Thus, transfer of mitochondria with intact mtDNA to cancer cells increases mtDNA copy number and promotes OXPHOS to support proliferation and metastasis (Mohammadalipour et al., 2020). Mitochondrial OXPHOS, biogenesis and respiration, as well as ROS levels, elevated as a consequence of mtDNA mutations, are important for motility of cancer cells and ensuing metastasis (LeBleu et al., 2014; Ishikawa et al., 2008).

**Figure 3.**
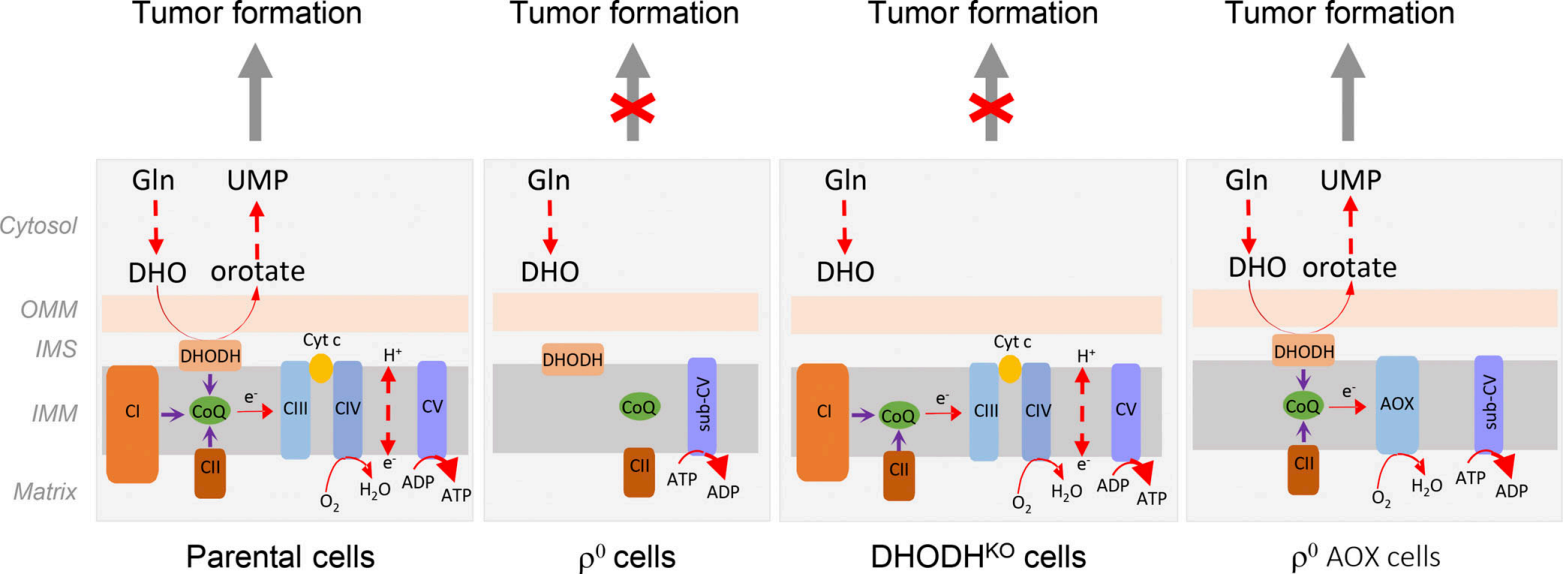
**Scheme of oxidative phosphorylation and its link to de novo pyrimidine synthesis and tumor formation.** In cells with functional mitochondria, electrons are fed into the ETC by CI and CII, and by DHODH, which catalyzes conversion of dihydroorotate (DHO) to orotate in the fourth reaction of de novo pyrimidine synthesis. The electrons are intercepted by the oxidized form of CoQ, which is reduced and carries the electrons to CIII and CIV. CoQ is re-oxidized to accept more electrons from the entry points. This includes DHODH, which allows for de novo pyrimidine synthesis to occur, so that cells can transit the S-phase and eventually undergo cytokinesis, facilitating tumor initiation and progression. In cells devoid of mtDNA (ρ^0^ cells), respiration is completely inhibited, so that DHODH does not function, de novo pyrimidine synthesis is halted, and tumors cannot develop or progress. DHODH^KO^ cells with functional respiration cannot transit the S-phase of the cell cycle since DHODH is inhibited. Restoration of the function of CIII and CIV, for example, by transfecting ρ^0^ cells with alternative oxidase (AOX), results in redox-cycling of CoQ, which restores the DHODH activity so that tumors can form and progress. Modified from Bajzikova et al. (2019). OMM, outer mitochondrial membrane; IMS inter-membrane space; IMM, inner mitochondrial space; UMP, uridine monophosphate.

Our discovery that cancer cells lacking mtDNA acquire mitochondria from stromal cells to promote cancer progression (Tan et al., 2015) can be reconciled with a study showing that cancer cells responsible for tumor relapse rely on OXPHOS for survival (Viale et al., 2014). Using time-resolved analysis of tumor formation by mtDNA-depleted cells and genetic manipulation of OXPHOS, we showed that de novo pyrimidine biosynthesis, dependent on respiration-linked DHODH activity, is required to overcome cell-cycle arrest. Thus, DHODH-driven de novo pyrimidine biosynthesis is an essential anabolic pathway coupling respiration with tumorigenesis (Bajzikova et al., 2019). This points to mitochondrial respiration being crucial for cancer cell proliferation, while energy is supplied primarily via glycolysis. The proposed link between mitochondrial respiration and de novo pyrimidine synthesis is depicted in Fig. 3.

## Therapeutic approaches and implications of horizontal transfer of mitochondria

HMT contributes to the rescue of mitochondrial function in recipient cells. It is a “double-edged sword” that provides both benefit and harm to cells and tissues, depending on the context. Its therapeutic implications are also twofold. HMT is detrimental in cancer where new mitochondria can rejuvenate damaged cancer cells and promote tumorigenicity, thus therapeutical benefit will be provided by inhibition of the process. In situations where new mitochondria enable normal tissue homeostasis, tissue stress resistance, or wound healing, therapeutic benefit will be provided by increased HMT. While pharmacological intervention targeting HMT is in an experimental stage, we outlined approaches with potential therapeutical relevance that imply mitochondrial transfer. The priority in this context is to identify molecular targets allowing selective modulation of HMT without compromising physiology of mitochondria and the cytoskeleton. An overview of possible therapeutic implications of mitochondrial transfer is in Table 3.

**Table 3.** Pathologies where mitochondrial transfer (or its blockage) has therapeutic impact

Pathology	Experimental model	Route of administration*	Result	Reference
Heart				
Ischemia	Heart regional ischemia	Injection into ischemic region	Enhanced myocardial functional recovery and cell viability	McCully et al., 2009
	Heart regional ischemia	Injection into ischemic region	Enhancement of post-ischemic myocardial function	Masuzawa et al., 2013
	Heart global ischemia	Coronary artery injection	Enhancement of post-ischemic myocardial function	Cowan et al., 2016
	Heart regional ischemia	Injection into ischemic region	Enhancement of myocardial cell viability	Kaza et al., 2017
	Heterotopic heart transplantation	Coronary artery injection	Enhancement of graft function and attenuation of necrosis	Moskowitzova et al., 2019
	Warm global ischemia	Coronary artery injection	Enhancement of post-ischemic myocardial function	Doulamis et al., 2020
	Heart regional ischemia	Pre-ischemic coronary artery injection	Enhancement of post-ischemic myocardial function	Guariento et al., 2020
Liver				
Ischemia	Partial liver ischemia	Intrasplenic injection	Attenuation of hepatic injury	Lin et al., 2013
	Non-alcoholic fatty liver disease	Intravenous tail injection	Attenuation of lipid accumulation and oxidative stress	Fu et al., 2017
	Acetaminophen-induced liver injury	Intravenous tail injection	Attenuation of tissue injury and enhancement of hepatocyte metabolism	Shi et al., 2018
Lungs				
	Experimental lung silicosis	Intravenous injection of MSCs or MSC-derived exosomes	Reduction in the size of silicotic nodules, total number of white blood cells in BALF, and expression of inflammatory and pro-fibrotic genes in the lung	Phinney et al., 2015
	Acute lung ischemia-reperfusion	Pulmonary artery injection and nebulization	Improvement of lung mechanics and attenuation of tissue injury	Moskowitzova et al., 2020
	Pulmonary fibrosis	Intravenous tail injection of MSCs	Mitigation of fibrotic progression	Huang et al., 2021
Kidney				
	Renal artery stenosis	Intra-arterially injection	Improved perfusion and oxygenation, protective effects on injured tubular cells	Zou et al., 2018
Diabetic nephropathy	Streptozotocin-induced diabetic rats	Injection under renal capsule	Improved cellular morphology and structure of the tubular basement membrane and brush border	Konari et al., 2019
CNS				
Stroke	Middle cerebral artery occlusion	Intravenous injection	Decrease of brain infarction area and partial neurological status restoration	Babenko et al., 2015
	Middle cerebral artery occlusion	Injection into ischemic striatum	Attenuation of brain infarction area and neuronal death, restoration of motor performance	Huang et al., 2016
	Middle cerebral artery occlusion	Intravenous injection	Restoration of neural function	Babenko et al., 2018
	Middle cerebral artery occlusion	Intra-arterial injection of MSCs	Improved mitochondrial function in peri-infarct area and functional recovery	Wang et al., 2019
	Middle cerebral artery occlusion	Intracerebro-ventricular injection	Promotion of neuroprotection, reduced brain infarct size, induced neurogenesis	Zhang et al., 2019
PD	Neurotoxin 6-hydroxydopamine induced PD	Medial forebrain bundle injection	Attenuation of oxidative damage and degeneration of dopaminergic neurons, and improved locomotion	Chang et al., 2016
	Neurotoxin MPTP induced PD	Intravenous injection	Reduction of neuronal death and attenuation of damage by ROS and improved behavioral symptoms	Shi et al., 2018
Schizophrenia	Poly-I:C induced schizophrenia	Prefrontal cortex injection	Prevention of the loss of brain ∆ψm and attention deficit in adulthood	Robicsek et al., 2018
AD	AD model produced by the intra-cerebro-ventricular injection of Aβ peptide	Intravenous injection (tail)	Attenuation of neuronal loss and reactive gliosis, restoration of cognitive deficits	Nitzan et al., 2019
Depression	LPS-induced model of depression	Intravenous injection	Improved symptoms such as exploratory behavior and promotion of neurogenesis, antidepressant-like effects	Wang et al., 2019
Aging	Aged mice (18 mo)	Intravenous injection (tail)	Significant improvement of cognitive and motor performance of aged mice	Liu et al., 2019
Spinal cord	Spinal cord injury	Mediolateral gray matter of injury site	Maintenance of acute bioenergetics, functional recovery	Zhao et al., 2020
	Spinal cord ischemia	Intravenous injection (jugular)	Improved hindlimb motor function	Feng et al., 2019; Gollihue et al., 2018
	Spinal cord injury	Injected into the epicenter of the injured spinal cord	Improved locomotor functional recovery	Li et al., 2019
Glaucoma	Optic nerve crush	Intravitreal injection	Promoted short-term neuroprotection (14 d) to retinal ganglion cells and modulated retinal oxidative metabolism; importantly, mitochondria also increased the number of axons extending ahead of the injury site in a long-term period (28 d)	McCully et al., 2017
Retinal ganglion cell degeneration	*Ndufs4* knockout mouse model	Vitreous cavity injection	Protection against mitochondrial damage-induced retinal ganglion cell loss	Jiang et al., 2019
Corneal injury	Alkaline burn-induced corneal damage	Transplantation of MSC scaffold to the center of the cornea	Improved corneal wound healing	Jiang et al., 2016
Cancer				
	Melanoma lung metastasis	Intravenous tail injection	Retardation of tumor growth and prolonged animal survival	Fu et al., 2019
	MM	Intravenous injection of CD38 myeloma cells	Targeting of CD38 reduced significantly mitochondrial transfer and improved animal survival	Marlein et al., 2019
	Glioma cell (U87) xenograft tumors	Injection into xenograft	Inhibited glioma growth, enhanced radiosensitivity of gliomas	Sun et al., 2019
Embryonic development	In vitro blastocyst stage development	Injection into zygotes	Improved embryonic development	Yi et al., 2007
Tissue injury	Full-thickness cutaneous wound and dystrophic skeletal muscle	Engraftment of MSCs and platelets into the wound area	Enhanced therapeutic efficacy of MSCs	Levoux et al., 2021
BM transplantation (BMT)	Total body irradiation as a preconditioning mechanism before BMT	Intravenous tail injection of BM cells	Rapid recovery of BM microenvironment, improved hematopoietic reconstitution after BMT	Golan et al., 2020

* administration of mitochondria unless stated otherwise

### Therapeutic approaches in cancer

The primary goal of HMT-focused therapy in cancer is to curb tumorigenicity and resistance associated with mitochondrial fitness of cancer cells (Berardi and Fantin, 2011; Suomalainen, 2019; Wei et al., 2019). Cancer cells can remove damaged mitochondria and transfer them to somatic cells, while healthy mitochondria can move in the opposite direction to “fix” cancer cells. The possible approaches include (i) interference with actin filaments in order to disrupt TNTs, (ii) interference with components required for TNT formation, (iii) reducing cell adhesion to lower transfer by limiting the time two cells spend in close proximity, and (iv) suppression of signals that stimulate HMT.

Pharmacological targeting of HMT may be applied in hematologic malignancies since HMT contributes to treatment resistance. Inhibition of the adhesion molecule ICAM-1 and treatment with cytochalasin D, an inhibitor of actin polymerization that disrupts TNTs, prevents mitochondrial transfer from MSCs to leukemia cells, promoting chemotherapy-induced cell death (Wang et al., 2018). Application of microtubule/actin inhibitors (vincristine, nocodazole, latrunculin B) reduces resistance to therapy (Burt et al., 2019). Suppression of ROS production reduced HMT from BM stem cells to acute myeloid leukemia blasts stimulates apoptosis and improves survival (Marlein et al., 2017). Similar reduction was observed in multiple myeloma (MM) upon TNT disruption by cytochalasin D as well as after blocking of CD38, a surface glycoprotein that stimulates TNT formation (Marlein et al., 2019). Targeting CD38 by monoclonal antibodies has been explored as a therapeutic strategy in leukemias, but it is unclear if inhibition of HMT contributes to its benefits (Koundinya et al., 2018; Martin et al., 2019; Moreno et al., 2019). During chemotherapy, including the use of the BH3 mimetic venetoclax, MM cells acquire healthy mitochondria from BM-derived MSCs via TNTs, and damaged mitochondria are mobilized from MMs by TNTs and MVs to be disposed of by MSCs (Matula et al., 2021).

Pharmacological targeting of HMT has been pursued in solid tumors. Breast/lung cancer cells hijack mitochondria from tumor-resident immune cell via TNTs, dampening the anti-tumor immune response and reducing the effectiveness of immune checkpoint therapy. Pharmacological inhibition of TNT formation in syngeneic subcutaneous mouse models of lung and breast cancer by the farnesyl/geranyl-1 transferase inhibitor L-778123 (inhibits Ras/Rho GTPase activity required for TNT formation) enhanced the efficacy of immune checkpoint blockade, restored immune response, and limited tumor growth (Saha et al., 2022). HMT was observed in a mouse model of GBM undergoing radiotherapy (Osswald et al., 2015). This supports the view that interference with HMT may be used as an approach for hard-to-treat tumors and lower their resistance to therapy (van Solinge et al., 2022).

### Therapeutic approaches in non-cancerous pathologies

In non-cancerous pathologies HMT is mostly beneficial. HMT improves the outcome of neurological injury and degenerative diseases affecting the brain, spinal cord, and visual system (Mohammadalipour et al., 2020), cardiovascular diseases including ischemia and cardiomyopathy (Han et al., 2016; Zhang et al., 2016), lung injury and asthma (Islam et al., 2012; Ahmad et al., 2014; Yao et al., 2018), wound healing (Levoux et al., 2021), and immune system function (Jackson et al., 2016a; Jackson et al., 2016b; Phinney et al., 2015; Morrison et al., 2017). The main objective is thus to stimulate HMT. This is a challenging task as pharmacological stimulation is usually more difficult than its inhibition. Researchers have tried to circumvent this issue, for example, by supplying healthy mitochondria to be incorporated into targeted cells, resorting to the so-called “mitotherapy.”

The therapeutic potential of mitotherapy was first explored in the cardiovascular system during ischemia/reperfusion injury. Respiration-competent mitochondria, isolated from healthy cardiac tissue, injected directly into the ischemic area of rabbit hearts during early reperfusion, enhanced myocardial functional recovery and cell viability (McCully et al., 2009). Autologous local transplantation of isolated mitochondria protected CMs from ischemia-reperfusion injury (Masuzawa et al., 2013) and offered protection during prolonged cold ischemia prior to heart transplantation (Moskowitzova et al., 2019; Moskowitzova et al., 2020). Infusion of mitochondria in the coronary artery reduced the infarct size and improved heart function (Guariento et al., 2020; Louwagie et al., 2021; Doulamis et al., 2020), and this procedure was applied in pediatric patients with congenital heart disease (Emani and McCully, 2018). It was shown that in response to energetic stress, adipocytes release ECVs containing respiration-competent mitochondria that are taken up by CMs, where they induced transient mitochondrial oxidative stress leading to pre-conditioning that protects against ischemia/reperfusion injury. This “‘metabolic pre-conditioning” was recapitulated by injection of adipocyte-derived ECVs (Crewe et al., 2021), and transplantation of mitochondria affected CM bioenergetics (Ali Pour et al., 2020). As mitotherapy also improves outcome in animal models of ischemia/reperfusion in the liver and lung (Moskowitzova et al., 2019; Moskowitzova et al., 2020; Lin et al., 2013), and in liver damage due to non-alcoholic fatty liver disease or exposure to toxic compounds (Fu et al., 2017; Shi et al., 2018), it can be concluded that mitotherapy by isolated mitochondria or mitochondria-containing ECVs has the potential to provide clinical benefit.

Mitotherapy was also extensively explored in the central and peripheral nervous system. Mitochondria derived from hamsters administered either via local intracerebral or systemic intraocular-arterial injection attenuated the area of brain infarction and neuronal death, and restored motor performance in a stroke model of middle cerebral artery occlusion (Huang et al., 2016). Administration of mitochondria by intra-cerebroventricular injection gave similar results, reducing reactive astrogliosis (Zhang et al., 2019). In a model of spinal cord injury exogenous mitochondria contributed to the maintenance of acute bioenergetics as well as functional recovery (Gollihue and Rabchevsky, 2017). In a model of Parkinson’s disease (PD), delivery of mitochondria into the medial forebrain attenuated the oxidative damage and degeneration of dopaminergic neurons, improving locomotion (Chang et al., 2016). In a model of schizophrenia, injection of mitochondria into the pre-frontal cortex of young rats prevented the loss of mitochondrial potential of brain cells and attention deficit in adulthood (Robicsek et al., 2018).

Besides mitotherapy, there are indirect approaches to stimulate HMT into damaged cells by delivery of efficient mitochondrial donors, such as MSCs that, once in close proximity, transfer mitochondria to recipient cells by ECVs or TNTs. Application of induced pluripotent stem cell-derived MSCs resulted in Miro1-dependent transfer of mitochondria, rescuing anthracycline-exposed CMs (Zhang et al., 2016). Administration of MSCs into lungs improved the outcome of LPS- and oxidative stress–induced lung injury (Islam et al., 2012; Li et al., 2018), allergic inflammation (Ahmad et al., 2014), and asthma (Yao et al., 2018). Mitochondria mobilized from mesenchymal stromal cells have beneficial effect on β cells, linking HMT and the metabolic syndrome (Rackham et al., 2020). Promising results of HMT were also observed in a model of AD (Nitzan et al., 2019) and wound healing (Levoux et al., 2021).

That HMT has a wide range of applications can be epitomized by two recent studies. Iron oxide nanoparticles enhanced formation of Cx43-containing gap junctions and increased the rate of HMT from administered MSCs in a model of pulmonary fibrosis (Huang et al., 2021); and exposure to hyperbaric oxygen led to amelioration of inflammation involving HMT (Lippert and Borlongan, 2019).

## Conclusions and future directions

Since the discovery of HMT in vitro (Rustom et al., 2004) and in vivo in mouse models of lung damage (Islam et al., 2012; Ahmad et al., 2014), and in tumor models where we used mtDNA polymorphisms and somatic cells from transgenic mice with RFP-decorated mitochondria (Tan et al., 2015; Dong et al., 2017), research on HMT has gained momentum. Initial in vivo research focused on the role of HMT in cancer and its functional consequences (Tan et al., 2015; Bajzikova et al., 2019) and possible modulation of resistance to cancer therapy (Osswald et al., 2015; Moschoi et al., 2016), and later on regulation of cancer-related immune responses (Saha et al., 2022). Role of HMT has been reported for a wide array of (patho)physiological conditions such as cardiovascular diseases, metabolic syndrome–related pathologies, and wound healing (Nicolas-Avila et al., 2020; Crewe et al., 2021; Levoux et al., 2021; Rackham et al., 2020). It is likely that HMT also occurs under natural conditions, as shown for outbred mice, with the idea being that regular exchange of mitochondria between cells contributes to maintaining balanced heteroplasmy (Jayaprakash et al., 2015), or during mouse development (Marti Gutierrez et al., 2022), which is important for homeostasis (Jackson et al., 2020) that may involve mitochondrial respiration-linked remodeling (Bennett et al., 2022). More research is needed to explore the role of HMT as a process in normal development and tissue homeostasis, in particular regarding the control of mtDNA heterogeneity. This is complicated because the vast majority of experimental animals are inbred with very limited mtDNA heteroplasmy.

Heterogeneity, mutations, and deletions in mtDNA have been implicated in a wide range of diseases, frequently involving neurodegenerative/neuromuscular pathologies (Burté et al., 2015; Lightowlers et al., 2015; Area-Gomez et al., 2019; Monzio Compagnoni et al., 2020; Ng et al., 2021). It is plausible that mitochondrial therapy, by stimulation of HMT or by mitochondrial transplantation, may be effective in alleviating pathologies, with neurodegenerative or cardiovascular diseases being “hot” candidates for the emerging therapeutic modality (Emani and McCully, 2018; Picard et al., 2016; Caicedo et al., 2017; Chang et al., 2019; McCully et al., 2017; Nascimento-Dos-Santos et al., 2021; Gorman et al., 2018). Mitochondrial transplantation has been used to replace faulty maternal mitochondrial genes (Gorman et al., 2018; Jacoby et al., 2022). We have witnessed considerable progress in mitotherapy based on mitochondrial transplantation including sophisticated approaches such as transfer of mitochondria from fetal to adult MSCs using optical tweezers (Shakoor et al., 2021). Mitotherapy has been boosted by the discovery of the so called MitoCeption that allows for controlled delivery of mitochondria isolated from donor cells (Caicedo et al., 2015; Sercel et al., 2021).

Faster implementation of mitotherapy, in particular based on HMT, is hindered due to methodological limitations. Unequivocal evidence for acquisition of mitochondria in vivo was provided by stable homoplasmic polymorphisms in mtDNA (Tan et al., 2015), known to occur in inbred mouse tumor models (Bayona-Bafaluy et al., 2003). Another problem in studying the molecular mechanisms and regulation of HMT is its visualization. This is now more facile by cutting-edge microscopic techniques, such as total internal reflection fluorescence microscopy (Henrichs et al., 2020), iterative tomography (Wu et al., 2021a), or multiview confocal super-resolution microscopy (Wu et al., 2021b), in particular in combination with genetically encoded fluorescent proteins decorating mitochondria (Barrasso et al., 2018).

Almost 20 yr after the discovery of mitochondrial transfer in vitro (Rustom et al., 2004) and <10 yr after the first unequivocal report of HMT in mouse tumor models (Tan et al., 2015), there is considerable momentum indicating that this very interesting and paradigm-shifting understanding of basic cell biology will be translated into therapeutic strategies that will alleviate multiple hard-to-treat pathologies. Of these, mitochondrial diseases linked to pediatric maladies and neurological pathologies are likely to be early candidates. We anticipate that what started as intriguing basic research will soon be translated for the benefit of mankind.
